# Formulation, pharmacokinetics, and antibacterial activity of florfenicol-loaded niosome

**DOI:** 10.1007/s13346-023-01459-9

**Published:** 2023-11-13

**Authors:** Shimaa G. Abonashey, Hatem A. F. M. Hassan, Mostafa A. Shalaby, Amr Gamal Fouad, Elham Mobarez, Hossny A. El-Banna

**Affiliations:** 1 Department of Biochemistry, Animal Health Research Institute, Dokki, Giza, Egypt; 2 Department of Pharmaceutics and Industrial Pharmacy, Faculty of Pharmacy, Cairo University, Cairo, 11562 Egypt; 3 School of Life and Medical Sciences, University of Hertfordshire Hosted By Global Academic Foundation, New Administrative Capital, Cairo, Egypt; 4 Pharmacology Department, Faculty of Veterinary Medicine, Cairo University, Cairo, Egypt; 5 Department of Pharmaceutics and Industrial Pharmacy, Faculty of Pharmacy, Beni-Suef University, Beni-Suef, Egypt

**Keywords:** Florfenicol, Niosome, Antibacterial activity, Antibiotics, Nanoparticles, Oral delivery

## Abstract

The growing interest in employing nano-sized pharmaceutical formulations in veterinary medicine has prompted the exploration of the novel nanocarriers’ ability to augment the therapeutic outcome. In this study, we harnessed niosomes, spherical nanocarriers formed through non-ionic surfactant self-assembly, to enhance the therapeutic efficacy of the broad-spectrum antibiotic florfenicol. Pre-formulation studies were conducted to identify the optimal parameters for preparing florfenicol-loaded niosomes (FLNs). These studies revealed that the formulation that consisted of Span 60, cholesterol, and dihexadecyl phosphate (DDP) at a molar ratio of 1:1:0.1 exhibited the highest entrapment efficiency (%EE) and uniform size distribution. In vitro antibacterial testing demonstrated the niosomal capacity to significantly reduce florfenicol minimum inhibitory concentration (MIC) against *E. coli* and *S. aureus*. Pharmacokinetic profiles of free florfenicol and FLN were assessed following oral administration of 30 mg florfenicol/kg body weight to healthy or *E. coli*–infected chickens. FLN exhibited a substantially higher maximum plasma concentration (*C*_max_) of florfenicol compared to free florfenicol. Furthermore, FLN showed significantly higher area under the curve (AUC_0–t_) than free florfenicol as revealed from the relative bioavailability studies. Lethal dose (LD) 50 values for both free florfenicol and FLN exceeded 5 g/kg of body weight, indicating high safety profile. Assessment of mortality protection in mice against lethal *E. coli* infections showed the significantly higher capability of FLN to improve the survival rate (75%) than free florfenicol (25%). Collectively, these findings demonstrate the niosomal ability to improve the oral bioavailability as well as the antibacterial activity of the incorporated veterinary antibiotic florfenicol.

**Graphical abstract:**

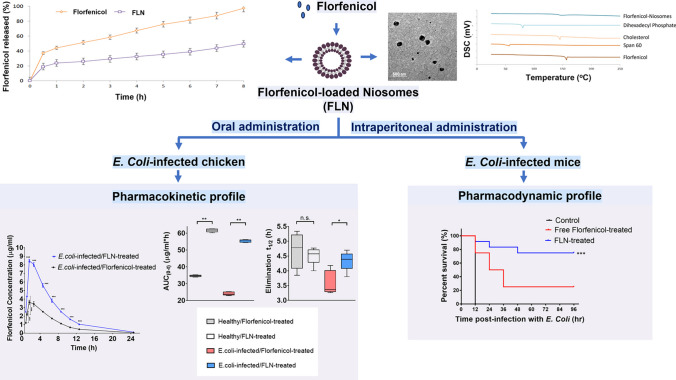

## Introduction

Florfenicol is a fluorinated derivative of thiamphenicol with a broad-spectrum antibacterial profile against both Gram-positive and Gram-negative bacterial strains. Florfenicol shares a common mechanism of antibacterial action with thiamphenicol and chloramphenicol [1]. Florfenicol belongs to the class of amphenicols that also includes chloramphenicol, thiamphenicol, and azidamfenicol. The classification within this group is established based on their shared phenylpropanoid structural motif [2, 3]. Florfenicol distinguishes itself as the only antibiotic within its class intentionally formulated for exclusive use in veterinary therapy. This choice stems from florfenicol’s capacity to mitigate the limitations associated with chloramphenicol, which had its approval revoked due to the manifestation of toxic secondary effects in humans through the consumption of animal-derived food products [1]. Florfenicol has demonstrated an acceptable safety profile and remarkable antibacterial efficacy at lower concentrations against enteropathogenic microorganisms, distinguishing it from its structural analogs, including thiamphenicol and chloramphenicol. Consequently, florfenicol is deemed an exemplary alternative to thiamphenicol and chloramphenicol [4]. This efficacy extends to a wide spectrum of pathogenic bacteria, such as *P. multocida*, *P. vulgaris*, *S. typhimurium*, *S. aureus*, *S. dysenteriae*, *K. pneumoniae*, *E. coli*, and *E. cloacae* [4]. The antibacterial efficacy of florfenicol can be attributed to its ability to hinder bacterial protein synthesis by selectively binding to ribosomal 50S and 70S subunits as well as the capacity to suppress bacterial enzymatic acetylation. [5]. Owing to its demonstrated effectiveness against chloramphenicol-resistant pathogens and its favorable safety profile characterized by low rates of adverse effects, florfenicol has found widespread utilization in veterinary clinics for the treatment of bacterial diseases [6–9].

Florfenicol was initially granted approval as a veterinary antibiotic for the treatment of cattle’s and pigs’ infection. Owing to its wide-ranging spectrum of activity, florfenicol has emerged as a valuable pharmaceutical agent in the management of prevalent diseases in broiler chickens, notably those caused by infection with *E. coli* [10]. Nonetheless, the oral administration of florfenicol is marked by diminished bioavailability, primarily attributed to its limited water solubility, a characteristic feature of class II drugs to which florfenicol belongs according to the Biopharmaceutical Classification System [11, 12].

The pharmacokinetic disposition of florfenicol was previously assessed in calves [13], lactating cows [14], and poultry [4, 10, 15]. Reportedly, the elimination half-life (*t*_1⁄2_) of florfenicol in poultry is less than 3 h that necessitates the administration of additional doses to achieve therapeutic effectiveness [4, 10, 15]. Thus, frequent dosing regimens are considered essential in clinical applications to augment therapeutic outcome [9]. However, it is worth noting that increased dosing frequency could lead to escalated labor costs and heightened stress levels in animals. To this end, unlocking the potential florfenicol’s therapeutic benefits necessitates the development of new and innovative formulations that enhance the therapeutic efficacy of florfenicol while reducing the dosing frequency [16, 17].

Nano-sized carriers have emerged as highly efficient tools capable of enhancing and modifying the pharmacokinetic and pharmacodynamic properties of drugs [18–21]. Previous studies have demonstrated nanoparticles’ potential to improve overall drug efficacy by enabling targeted and controlled drug delivery [18, 19, 22]. Niosomes, in particular, could offer higher stability when compared to commonly utilized self-assembled vesicular carriers, such as liposomes. This distinctive advantage can be attributed to the phospholipid composition of liposomes that is characterized by its high susceptibility to oxidation and hydrolysis [18, 19].

Niosomes can be described as nano-sized drug delivery systems primarily comprised of non-ionic surfactants and cholesterol [18, 19, 23–25]. Niosomal carriers have the potential to enhance drug accumulation at specific tissues, thereby maximizing therapeutic efficacy [19, 26]. Additionally, previous studies have highlighted the capacity of niosomes to enhance drug stability, reduce drug toxicity, prolong circulation time, and facilitate drug uptake at the target sites [18, 19, 23]. These distinctive features of niosomes hold the potential to potentiate the antimicrobial activity of loaded antibiotics, maintain therapeutic concentrations in the plasma, and ultimately reduce the required antibiotic dosage. Consequently, the niosomal formulation presented in this study represents an efficient approach to mitigate the propensity for antibiotic resistance establishment [27].

Recent pharmaceutical formulations utilized in florfenicol delivery, including nanoemulsions [1, 28] as well as polymeric nanoparticles composed of natural or synthetic polymers such as chitosan [29] or poly(lactic-co-glycolic acid) (PLGA) [30], respectively, hold significant promise for enhancing the florfenicol efficacy. These formulations offer unique attributes, including controlled release, stability, and targeted drug delivery. For instance, PLGA-based polymeric nanoparticles could provide sustained florfenicol release profile and facilitate targeted delivery to specific tissues. Nanoemulsions, on the other hand, could enhance the florfenicol’s solubility and bioavailability.

Nevertheless, the limitations and challenges associated with these innovative delivery systems, such as potential toxicity and stability issues, have to be properly recognized and assessed. These factors must be carefully considered and addressed to fully harness the nanoformulations’ potential in improving the therapeutic efficacy of florfenicol.

In this study, a novel nanoformulation was developed to address the inherent poor aqueous solubility of florfenicol, rendering it a viable therapeutic option for managing bacterial infections in poultry through oral administration. The niosomal florfenicol formulation was prepared and characterized. Moreover, comparative studies were carried out to evaluate the pharmacokinetic profiles of both free florfenicol and FLN in healthy and *E. coli*–infected broiler chickens after a single oral dose. Furthermore, we assessed the niosomal florfenicol formulation’s capacity to prolong the survival of mice infected with lethal *E. coli*.

## Materials and methods

### Materials

Florfenicol, Tween 60, Span 60, cholesterol, DDP, ethylene acetate, and acetonitrile were obtained from Sigma Aldrich (Cairo, Egypt). Chloroform and methanol were obtained from Corner-Lab Company (Egypt). Nylon 0.45-µm filter was obtained from Millipore (Massachusetts, US). *E. coli* (ATCC35218), *Sal. typhimurium* (ATCC14028), and *S. aureus* (ATCC29213) were obtained from Microbiological Resources Centre (Mircen, Cairo, Egypt).

### Pre-formulation study

Pre-formulation studies were conducted to determine the optimal FLN formulation conditions for subsequent in vitro and in vivo investigations. Various FLN formulations were prepared, with hydrophilic lipophilic balance (HLB) values ranging from 4.7 to 14.9, surfactant-cholesterol molar ratios spanning from 0.5 to 2, and DDP-surfactant molar ratios ranging from 0 to 0.4, serving as the independent variables [19, 31–33]. Non-ionic surfactants, namely, Span 60 and Tween 60, were employed in these formulations [18, 24, 33, 34]. The dependent variables considered were particle size and %EE. Formulation conditions that led to the highest %EE of florfenicol and the smallest niosome particle size were selected for the subsequent development of the FLN used in the further investigations.

### Preparation of FLN formulations

Using the thin-film hydration method, a range of FLN formulations were developed [35]. In a round-bottom flask, a 10-ml solution of chloroform and methanol, mixed at 3:1 (v/v), was prepared. This prepared solution contained 10 mg of florfenicol along with calculated quantities of Tween 60, Span 60, cholesterol, and DDP. Subsequently, the organic phase was evaporated under vacuum at 100 rpm and 40 °C using a Stuart rotary evaporator (RE300, UK). The resulting film was then reconstituted in a 10-ml phosphate buffer through incubation at 60 °C for 2 h. Each formulation underwent a cooling process in an ice-water bath to mitigate the risk of excessive heating, followed by the application of sonication using an ultrasonicator (Sonix, IL, USA) for 30 min. Sonication was applied in pulses, with a 50% amplitude, employing a sequence alternating between 2 s of sonication and 2 s of intermission. The resultant mixture was centrifuged at 15,000 rpm for 1 h to isolate FLN pellets that were subsequently diluted with 10 ml of phosphate buffer and stored at 4 °C.

### Optimization and in vitro characterization of FLN formulations

#### Determination of %EE

The assessment of florfenicol entrapment in the various FLN formulations was carried out by determining the %EE (Eq. 1) [26]. The prepared formulations were centrifuged at speed of 15,000 rpm at 4 °C for 1 h. The supernatant and yielded niosomes pellets were separated. Subsequently, the collected niosomes pellets were washed three times with PBS to remove un-entrapped florfenicol. After each wash, the mixture was centrifuged for an additional 1 h and all the supernatants were carefully collected. The supernatants were analyzed using a UV/Vis spectrophotometer at 267 nm to quantify the unencapsulated florfenicol. The %EE was calculated in triplicate using the following equation:

1 $$\%EE= \frac{(\mathrm{Initial\;florfenicol\;amount}-\mathrm{ The\;amount\;of\;florfenicolin\;the\;supernatant})}{\mathrm{Initial\;florfenicol\;amount}} \times 100$$

#### Particle size, polydispersity index (PDI) and zeta potential determination

Niosomal characteristics that could impact particle dispersion, uniformity, distribution, and consequently, their deliverability, such as particle size and polydispersity index (PDI), were assessed [23]. To evaluate surface properties and stability, the zeta potential of FLN nanoparticles was determined to measure their surface charge [24, 32]. In brief, a 1:10 dilution was prepared by mixing 1 ml of the niosomal florfenicol formulation with 9 ml of distilled water; subsequently, the particle size and PDI of the prepared formulations were measured in triplicate using dynamic light scattering (DLS, Malvern, UK) [36].

#### Differential scanning calorimetry (DSC)

FLN mixture was centrifuged at 15,000 rpm for 1 h to isolate FLN pellets that were then lyophilized using a freeze dryer (EF03, Edwards High Vacuum Ltd.) DSC analysis was conducted on florfenicol, the lyophilized optimal FLN formulation, and its individual components, specifically Span 60, cholesterol, and DDP [37]. Samples weighing between 3 to 5 mg were carefully loaded to DSC aluminum pans with a thickness of 0.1 mm. DSC thermograms were acquired utilizing a DSC analyzer (60F3, Maia, Germany) operating within a temperature range spanning from 20 to 300 °C, employing a heating rate of 5 °C/min, and maintaining a flow of nitrogen gas at 25 ml/min.

#### Transmission electron microscopy (TEM)

A 20 µl of the optimal FLN formulation was deposited onto a carbon-coated copper grid and subsequently stained with phosphotungstic dye [38]. The resulting FLN structures were examined for their morphological characteristics at various magnifications using TEM (Carl Zeiss, Germany).

#### Release kinetics in vitro

The release of niosome-incorporated florfenicol was assessed following a previously reported method [38]. A dialysis bag was pre-incubated in phosphate-buffered saline (pH 7.4) supplemented with 0.1% Tween 80 for a duration of 24 h. Subsequently, the dialysis bag was loaded with 2 mg of free or niosome-encapsulated florfenicol. The filled dialysis bag was then immersed in a beaker containing 50 ml of phosphate-buffered saline solution supplemented with 0.1% Tween 80 that served as the medium for drug release. Assessment of florfenicol release in vitro was performed utilizing the Hanson dissolution apparatus at 37 °C with continuous stirring at a rate of 100 rpm. At various time intervals, a 3 ml sample was removed and replenished with fresh medium to keep the sink condition. The released florfenicol was quantified using a spectrophotometer set to a wavelength of 267 nm to determine the cumulative release versus time (h). The kinetic analysis of the release data was performed using DDSolver program [38]. A model matrix of forty models was employed in the evaluation of the best-fitted model based on the coefficient of determination (R^2^), the Akaike information criterion (AIC), and the model selection criterion (MSC). The model with the lowest AIC and highest R^2^ and MSC closely corresponded to the drug release profile. The release exponent "n" of the Korsmeyer-Peppas equation was calculated to determine the drug's release mechanism.

### Determination of MIC

The MIC of free or niosome-encapsulated florfenicol as well as florfenicol mixed with an equivalent concentration of cholesterol matching that used in the niosomal formulation was determined against *E. coli* (ATCC35218), *Sal. Typhimurium* (ATCC14028), and *S. aureus* (ATCC29213) in vitro through the broth macro-dilution procedure [39]. In brief, serial dilutions of free, niosome-incorporated or cholesterol-mixed florfenicol were prepared within the range of 0.031 to 32 µg/ml, using the respective micro-organism's culture media in sterile Mueller–Hinton broth. These dilutions were then transferred to test tubes containing fresh culture media with a final inoculum concentration of 1 × 10^5^ CFU/ml. The test tubes were subsequently incubated at 37 °C, and the MIC was defined as the lowest florfenicol concentration at which visible growth inhibition was observed after the 37 °C incubation period. All experiments were conducted in triplicate and repeated three times (*n* = 9).

### In vivo studies

#### Animals

All institutional and national guidelines governing the care and utilization of laboratory animals were rigorously adhered to and received the approval of the Institutional Animal Care and Use Committee (IACUC) at the Faculty of Veterinary Medicine, Cairo University, Egypt (Vet CU 01122022612).

#### Bacterial infection induction

A total of thirty clinically healthy Hubbard chickens, each 5 weeks old with a mean weight of 1.73 kg, were procured from private poultry farms situated in Giza, Egypt. To ensure the complete elimination of any residual drugs from their systems, the chickens were maintained on a balanced antibiotic-free diet for a duration of 2 weeks. During this period, the chickens were also exposed to stress-inducing conditions. The water and food provided to the chickens were devoid of any antibacterial additives. The chickens were then categorized into five groups, each consisting of six individuals (*n* = 6). Enteropathogenic *E. coli* O78 (3 × 10^8^ CFU/ml) obtained from the Bacteriology Department, Animal Health Research Institute (Giza, Egypt), was inoculated on MacConkey broth and incubated at 37 °C for 2 days. The bacteria were then sub-cultured on MacConkey agar and incubated at 37 °C for a day and the obtained culture was plated on CFU agar plate. Three groups served as the *E. coli–*infected group*,* while the other two groups remained uninfected and served as the healthy control groups. Chicken infection was carried out via the oral inoculation with 1 ml of saline containing 10^8^ CFU *E. coli*/ml [40]. After the inoculation, the chickens were placed under observation for pathological signs and symptoms such as diarrhea, loss of appetite, and ruffled feathers. Positive bacterial cultures in MacConkey broth were used to confirm the presence of clinical symptoms and successful infection with *E. coli*. Accordingly, bacterial cultures were conducted on chicken liver, heart, and spleen tissues to assess clinical symptoms. These cultures were incubated in MacConkey broth at 37 °C for 1 day to isolate the *E. coli* O78 strain using its specific antiserum.

#### Oral administration of florfenicol and FLN

A single dose of free florfenicol (30 mg/kg body weight) was orally administered to control healthy broilers group (the healthy/florfenicol-treated group) and *E. coli*–infected broilers (the *E. coli*–infected/florfenicol-treated group). Simultaneously, FLN was orally administered at 30 mg florfenicol/kg of body weight to another control healthy group (the healthy/FLN-treated group) and *E. coli*–infected broilers (the *E. coli*–infected/FLN-treated group). Each group consisted of 6 subjects. At various designated time intervals following the oral administrations, blood samples of 1 ml each were collected through wing vein puncture into heparinized tubes. Subsequently, the blood samples were centrifuged at 4000 rpm for a duration of 10 min. The resultant clear serum samples were collected and preserved at − 20 °C until the commencement of assay procedures.

#### Florfenicol quantification in plasma samples

Florfenicol concentration in plasma samples was determined using high-performance liquid chromatography (HPLC) [41]. An Agilent HPLC system was employed for the separation and quantification of florfenicol. The optimal mobile phase was established using a mixture of acetonitrile and water in an 18:82 ratio, with a flow rate of 1 ml/min. Florfenicol concentration was detected by measuring the UV absorption at 224 nm. The mobile phase was filtered, degassed using 0.45-µm nylon filter under vacuum and was sonicated for 30 min. The flow rate was maintained at 1 ml/min and the injection volume used was 10 µl. Plasma samples were extracted using ethylene acetate (0.5 ml:1.25 ml). The collected tubes were centrifuged at 2000 g for 10 min. Afterwards, 1 ml of the organic layer was then aspirated and evaporated under nitrogen. The obtained residues were dissolved in 0.375 ml of solvent mixture that consisted of acetonitrile and water (1:2, v/v), vortexed and then centrifuged at 19,000 g for 20 min at 4 °C. The supernatant was collected, filtered through a 0.45-μm nylon filter, and finally transferred to auto-sampler vials. Florfenicol standards at various concentrations (0.05–5 µg/ml) were prepared. A correlation coefficient (*r*^2^) and accuracy of 0.99998 and 99.3 ± 1.36 were achieved, respectively. A calibration curve was constructed by plotting florfenicol peak areas against the known concentrations. The equation was determined through the least-squares method using linear regression. The pharmacokinetic parameters were analyzed using PK solver 2.0.

#### Acute toxicity

The method described by Wang et al. was applied [1]. Fifty mice, obtained from Animal Health Research Institute (Giza, Egypt), each weighing 25 g, were allocated randomly into five groups with each group consisting of five males and five females (*n* = 10). All mice underwent an overnight fasting period while having unrestricted access to water.

The FLN and free florfenicol powder employed in the acute toxicity studies was lyophilized and suspended in distilled water, respectively. As a negative control, the first group was orally administered with 500 μl of sterile distilled water. The second and third groups were orally administered with single dose of free florfenicol at 2 and 5 florfenicol g/kg body weight in 500 μl sterile distilled water, respectively. Animals in the fourth and fifth groups were orally administered with a single dose of FLN at 2 and 5 florfenicol g/kg body weight in 500 μl sterile distilled water, respectively. All mice were observed continuously for 7 days after administration for mortality and clinical symptoms.

#### Determination of bacterial infection lethal dose

A preliminary experiment was conducted to determine the lethal bacterial dose 100% (LD100) of *E. coli* (O78) in mice. Forty mice, obtained from Animal Health Research Institute (Giza, Egypt), each weighing 25 g, were randomly allocated into four groups (10 animals per group). Bacterial suspension (0.2 ml) was intraperitoneally injected into each mouse of the respective group at 1 × 10, 1 × 10^7^, 1 × 10^8^, or 1 × 10^9^ CFU/ml. The animals were observed every 6 h over a total period of 72 h. The *E. coli* concentration of 1 × 10^9^ CFU/ml proved lethal (LD100) as it resulted in the mortality of all the mice within the group, whereas mice in the other infected groups demonstrated survival. The determined lethal dose, 1 × 10^9^ CFU/ml of *E. coli*, was subsequently employed for mortality protection studies.

#### Mortality protection study

Seventy-two mice, obtained from Animal Health Research Institute (Giza, Egypt), each weighing 25 g, were randomly allocated to six groups. Each group consisted of 12 animals, including 6 males and 6 females. The first, second, third, fourth, and fifth groups were intraperitoneally injected with lethal inoculum size of *E. coli* (0.2 ml of 1 × 10^9^ CFU/ml). The seventh group served as uninfected control. The first group served as the infected and untreated control negative group, with each mouse receiving an equivalent volume of sterile distilled water. The second and third groups were intraperitoneally injected with florfenicol in its free form at a single florfenicol dose of 20 and 40 mg/kg body weight in 0.2 ml sterile distilled water, respectively. Simultaneously, the fourth and fifth groups were intraperitoneally injected with a single dose of FLN, containing 20 mg/kg and 40 mg/kg of florfenicol in 0.2 ml sterile distilled water, respectively. The animals were regularly observed at 12-h intervals, and occurrences of mortality were recorded over a 96-h period [1].

### Statistical analysis

The obtained data were subjected to statistical analysis. These analyses were conducted utilizing the commercially available software packages SPSS Inc. version 20.0 (USA) and GraphPad Prism version 7.00 (USA), to express the differences between groups.

## Results and discussion

### Pre-formulation study

Various FLN formulations were prepared, and pre-formulation studies were conducted to assess the influence of independent variables on the yielded niosomes (Table 1) [19, 31–33]. The surfactant’s polarity, as expressed by the HLB value, was used as a guide in the selection of suitable surfactants to facilitate the production of physically stable niosomes [23, 24, 42]. Non-ionic surfactants, namely, Span 60 and Tween 60, were employed, as previous studies indicated that their extended alkyl chains could enable the formation of small-sized niosomes with a high %EE [18, 24, 33, 34]. The determined %EE of the prepared FLN formulations ranged from 65.72 ± 1.34 to 90.32 ± 0.97%, and the particle size ranged from 246.5 ± 9.65 to 542.76 ± 6.4 nm. The pre-formulation studies demonstrated that niosomes formulated using Span 60 exhibited the highest florfenicol encapsulation (*p* < 0.05) and the smallest particle size (*p* < 0.05). This observation could be attributed to the lipophilic nature of florfenicol, the low HLB value, and the diminished surface native energy of Span 60, which potentially promoted the formation of stable vesicles [18, 23, 24, 26, 33]. Furthermore, it was observed that an increase in HLB was associated with a decrease in %EE (*p* < 0.05) and an increase in the vesicle size (*p* < 0.05). This observation could be linked to the higher hydrophilicity and surface native energy of Tween 60 [33]. These findings were consistent with previously reported studies [23, 26].

**Table 1:** %EE and vesicle size of the different FLN formulations

**Formulation** **code**	**Non-ionic surfactant**	**Cholesterol to** **non-ionic surfactants ratio**	**DDP to** **non-ionic surfactant** **ratio**	**EE (%)**^**a**^	**Vesicle size (nm)**^a^
F1	Span 60	1:1	0	80.14 ± 0.66	340.14 ± 9.82
F2	Tween 60	1:1	0	65.72 ± 1.34	445.58 ± 6.25
F3	Span 60	1:1	1:10	90.32 ± 0.97	246.5 ± 9.65
F4	Tween 60	1:1	1:10	72.09 ± 1.05	385.13 ± 7.19
F5	Span 60	2:1	1:10	76.51 ± 0.85	361.30 ± 13.72
F6	Tween 60	2:1	1:10	82.77 ± 0.58	293.03 ± 6.25
F7	Span 60	1:1	2:10	85.48 ± 0.72	289.79 ± 5.18

^a^Results represent the mean value ± SD (*n* = 3)

Cholesterol, characterized by its inherent rigidity, could exert a discernible influence on niosomal structures and their physical attributes, including %EE, in vitro stability, payload release, and biostability [18, 23, 33, 42]. The incorporation of cholesterol could protect the formulated niosomes against destabilizing factors, including plasma and its constituents, while simultaneously mitigating the leakage of encapsulated molecules by reducing vesicular permeability [18, 23, 33, 42]. This could be assigned to cholesterol-mediated vesicular rigidity and stability. The findings of pre-formulation studies unveiled a notable correlation between cholesterol content, %EE, and particle size, with this relationship significantly dependent on the HLB value of the employed surfactant. The increase in the HLB along with the cholesterol content led to an increase in %EE (*p* < 0.05) and a reduction in niosomal particle size (*p* < 0.05). This could be a result of cholesterol’s capacity to diminish surface native energy, elevate bilayer hydrophobicity, and fortify rigidity, culminating in the formation of less permeable and more stable vesicles. Conversely, the reduction of HLB while simultaneously increasing cholesterol content within the formulation led to a decline in %EE (*p* < 0.05) and an increase in particle size (*p* < 0.05). This shift could be ascribed to structural perturbations within the niosomes induced by the added cholesterol and the impeding influence of surfactants at the vesicular membrane [32, 19, 33]. These findings align with previous studies reported by Chaw and Ah Kim [32] and Waddad et al. [33].

DDP, a charge inducer, was employed in the preparation of niosomes to impart a net negative zeta potential that could enhance the niosomal stability [24, 33]. The incorporation of DDP into the FLN formulations resulted in an increase in %EE and a reduction in particle size. Nevertheless, upon further incorporation of DDP, an inverse relationship was detected that was characterized by a decrease in %EE and an increase in particle size. This could be due to the DDP-mediated elevation in the niosomal surface native energy and enhancement of the bilayer hydrophilicity [24, 32, 33, 43, 44]. The optimization studies highlighted crucial formulation parameters that can be harnessed to maximize %EE and minimize particle size. The optimal formulation composition for niosome preparation that yielded high %EE (90.32 ± 0.97%) and a small particle size (229.3 ± 7.18 nm), consisted of Span 60, cholesterol, and DDP at a molar ratio of 1:1:0.1.

### Characterization of optimum FLN formulation

#### FLN particle size, zeta potential, and morphology

**Fig. 1:**
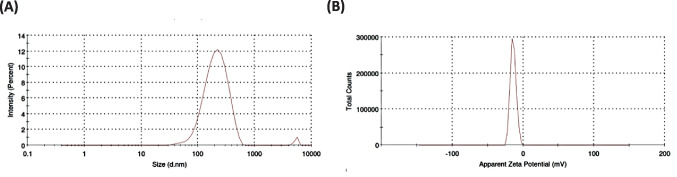
Particle size (**A**) and zeta potential (**B**) of the optimum FLN formulation

The optimal FLN formulation exhibited a reduced particle size with a low PDI of 0.258 that suggested a uniform size distribution, minimal interfacial tension, homogenous niosome formulation, and a diminished aggregation propensity [23, 24, 33]. The determined zeta potential of the optimal FLN formulation was − 19.50 ± 1.12 mV (Fig. 1). The negative surface charge observed in the FLN formulation indicated the presence of vesicular stability attributable to electrostatic repulsions between the vesicles [24, 32, 38, 45].

**Fig. 2:**
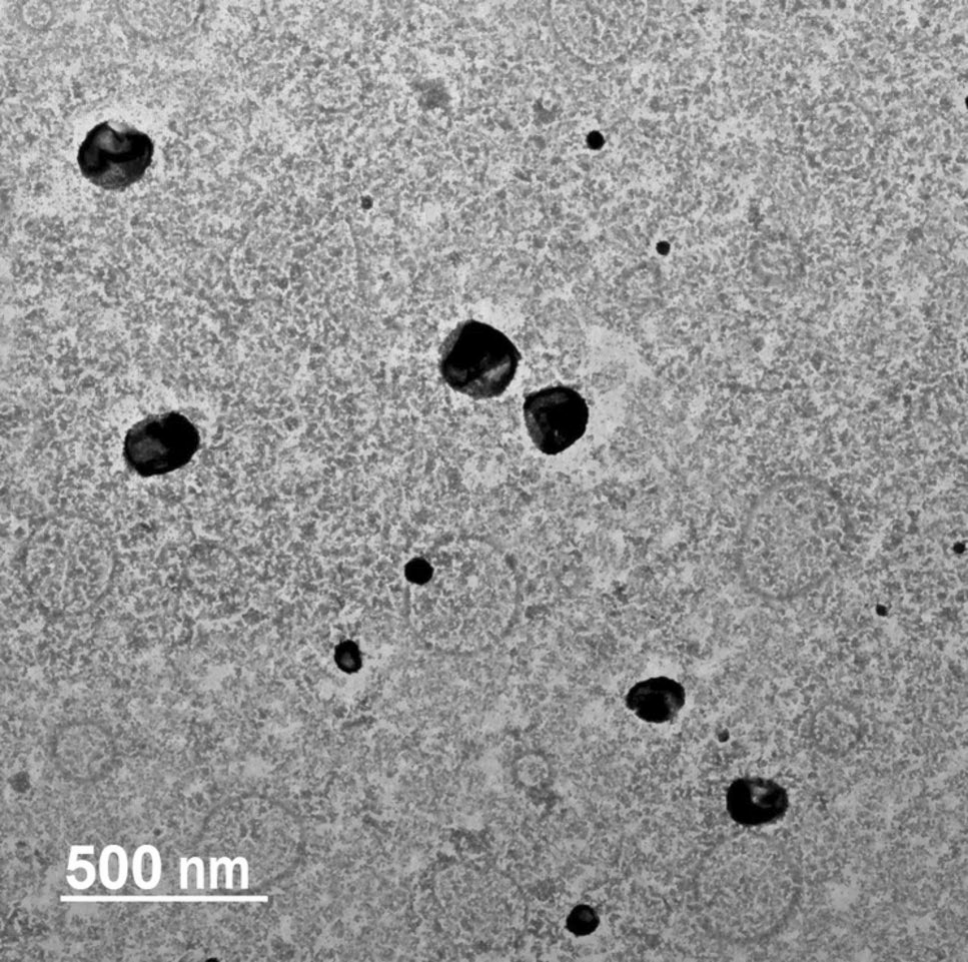
Morphological features of FLN formulation determined using TEM

The morphological features of the optimal FLN formulation were determined using TEM, as depicted in Fig. 2. The TEM images revealed the presence of individualized spherical vesicular structures characterized by well-defined outlines and absence of noticeable aggregations.

#### DSC

**Fig. 3:**
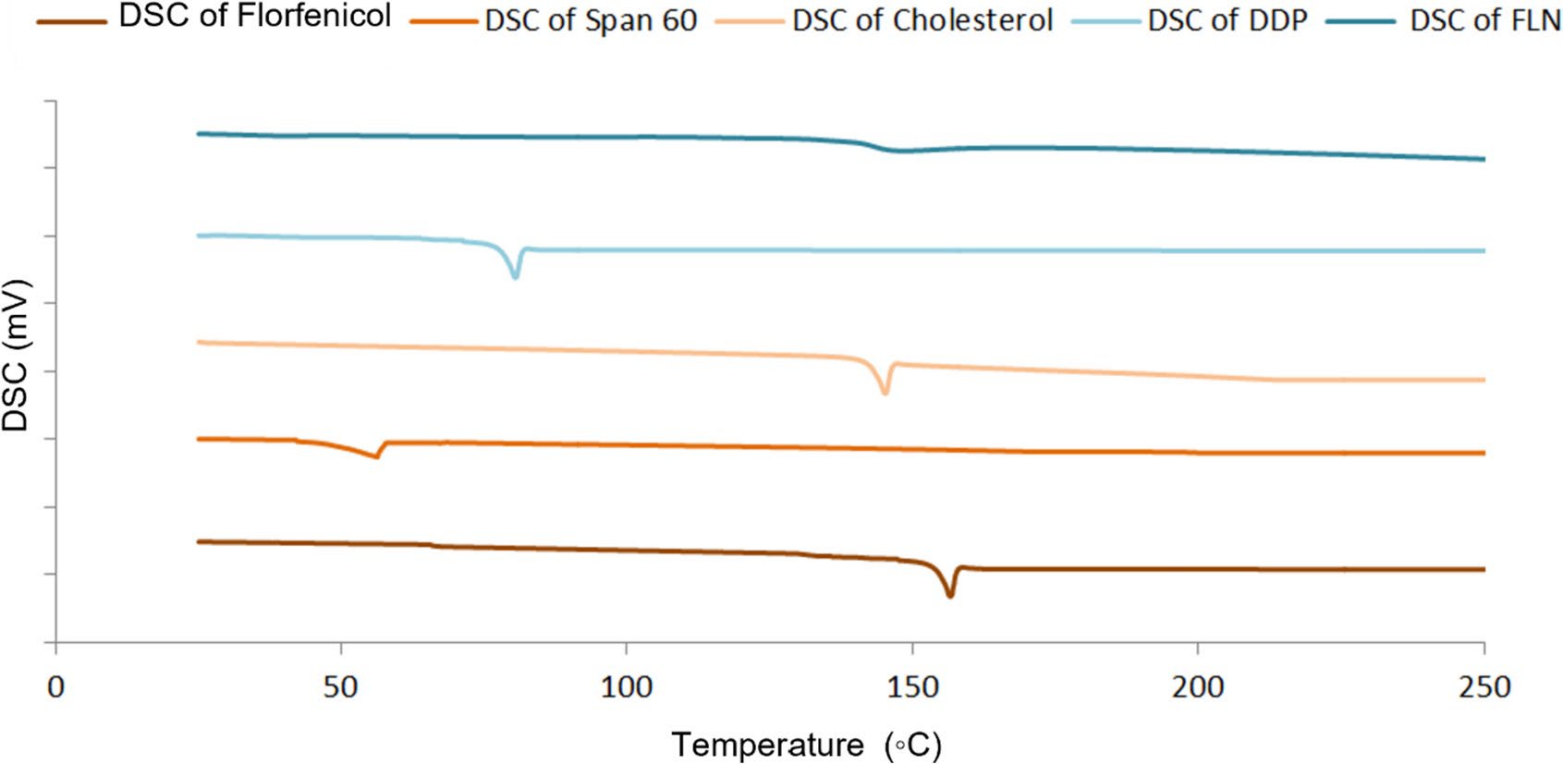
DSC thermograms of FLN formulation and its individual constituents, namely, pure florfenicol, Span 60, cholesterol, and DDP

DSC analysis was employed to elucidate the impact of the niosomal formulation on the thermodynamic properties of its constituents, including florfenicol, by altering their crystalline characteristics [46, 47]. The thermograms of the lyophilized optimal FLN formulation and its individual constituents, namely, Span 60, cholesterol, DDP, and pure florfenicol, are shown in Fig. 3. DSC thermograms of florfenicol, Span 60, DDP, and cholesterol exhibited distinct sharp endothermic peaks at 154.65 °C, 52.09 °C, 79 °C, and 149 °C, respectively, corresponding to their respective melting points. In contrast, the DSC thermogram of the optimal FLN formulation displayed an absence of these characteristic peaks. The reduced crystallinity observed in DDP, cholesterol, and Span 60 upon their mixture suggests the formation of bilayers. The diminished crystallinity of florfenicol may be attributed to its incorporation into the bilayer structure of self-assembled Span 60 in an amorphous state.

#### In vitro release kinetics

**Fig. 4:**
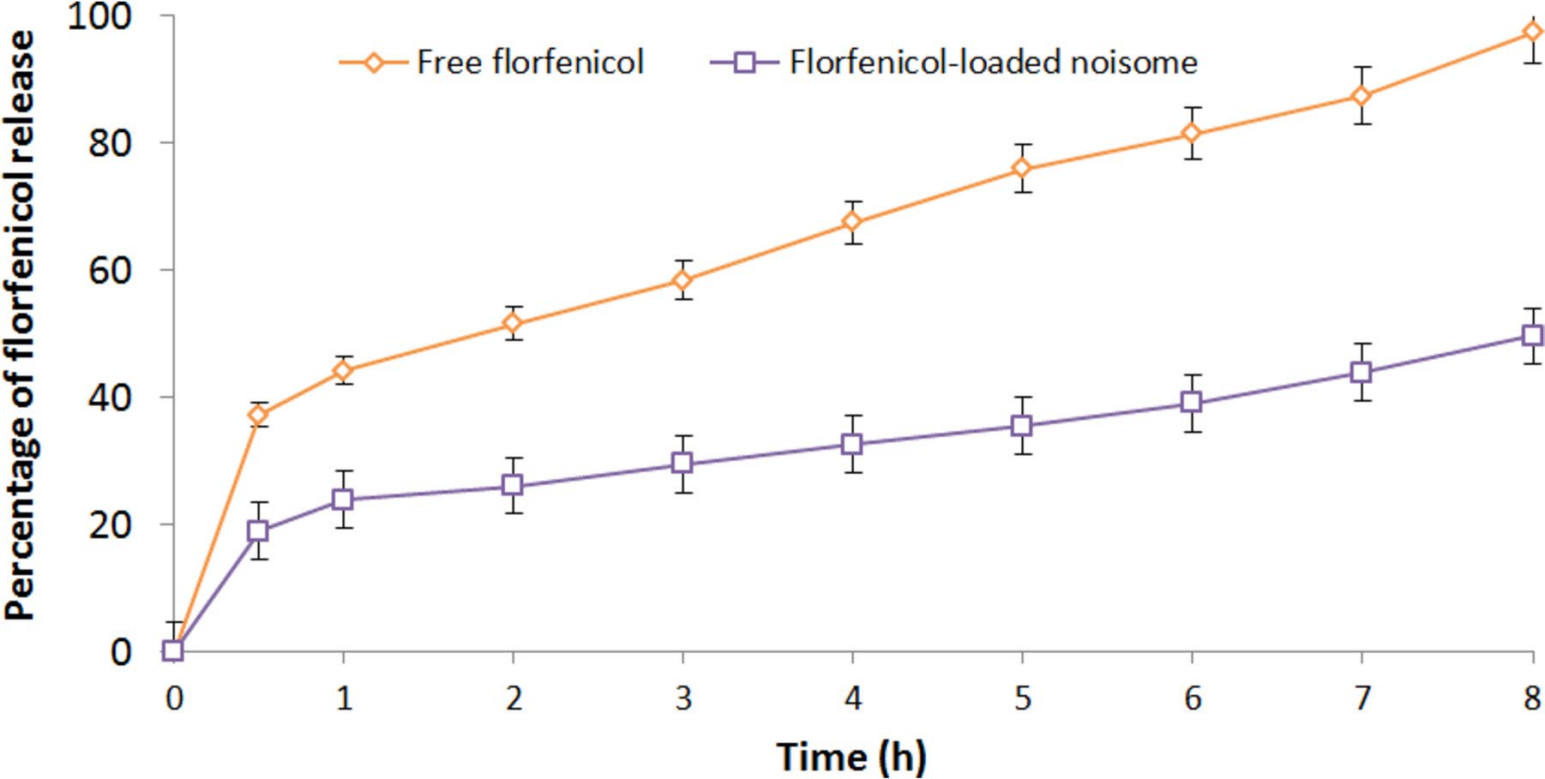
In vitro release profile of FLN formulation in comparison to free florfenicol

The in vitro release profile demonstrated that 97.42% of the free florfenicol was released in 8 h (Fig. 4). On the other hand, the FLN formulation released around 23.81% of its florfenicol content within the first hour, this was followed by a prolonged release profile, and 49.60% of the encapsulated florfenicol was released after 8 h. The delayed release could be attributed to the structural affinity of the niosome-encapsulated florfenicol with the alkyl chain of Span 60 as well as the incorporated cholesterol. The release kinetics results indicated that the Korsmeyer-Peppas model adequately described the release data fit with highest *R*^2^ of 0.967, lowest AIC of 44.1745, and highest MSC of 2.3678. The determined release exponent (*n*) was 0.363 that suggested Fickian diffusion release mechanism.

### In vitro antibacterial activity

The MIC of free or niosome-incorporated florfenicol was determined using the broth macro-dilution procedure. The bacterial strains employed included *E. coli* (ATCC 35218), *Sal. typhimurium* (ATCC 14028), and *S. aureus* (ATCC 29213). The obtained MIC values are summarized in Table 2. The MIC values against *E. coli* were 4 ± 0.0 μg/ml for free florfenicol and 0.5 ± 0.0 μg/ml for FLN. These findings indicated a substantial increase in the antibacterial activity of florfenicol when incorporated into niosomes. The increased antibacterial efficacy of the FLN formulation could be ascribed to the enhanced bacterial uptake of nanoparticles as well as the intrabacterial accumulation facilitated by the internalized niosomes [48–50]. In this context, Yang et al*.* suggested that the direct interaction between florfenicol-loaded nanoparticles and bacterial cell membranes led to the disruption of bacterial cell integrity that ultimately resulted in intrabacterial release of florfenicol [51]. On the contrary, the obtained result showed that MIC of free florfenicol and niosomes-contained florfenicol were 0.25 ± 0.0 and 1 ± 0.0 μg/ml against *Sal. typhimurium*, respectively. This indicated that the free florfenicol exhibited higher antibacterial activity than FLN against *Sal. typhimurium*. Interestingly, the addition of equivalent concentration of cholesterol to free florfenicol did not affect the MIC against *E. coli* and *S. aureus* but led to an increase in the MIC values against *Sal. typhimurium* (1 ± 0.0 μg/ml). It was previously demonstrated that the presence of cholesterol in the culture media resulted in an increase in the MIC of antibiotics such as ciprofloxacin, tetracycline, and chloramphenicol against *Sal. typhimurium*. This observation could be attributed to cholesterol-mediated reduced *Sal. typhimurium* sensitivity resulting from protective biofilm formation [52]. Thus, it could be suggested that the observed higher MIC for FLN compared to free florfenicol against *Sal. typhimurium* was linked to the cholesterol content of the former.

**Table 2:** MIC of florfenicol (μg/ml) against *E. coli*, S*. aureus,* and *Sal. typhimurium*

	MIC of florfenicol^a^ (µg/ml)	
Bacterial strain	Free florfenicol	FLN
*E. coli* (ATCC35218)	4 ± 0.0	0.5 ± 0.0
*S. aureus (ATCC29213)*	2 ± 0.0	0.25 ± 0.0
*Sal. typhimurium (ATCC14028)*	0. 25 ± 0.0	1.0 ± 0.0

^a^Results represent the mean value ± SD (*n* = 9)

### Pharmacokinetic profile of orally administered FLN

**Fig. 5:**
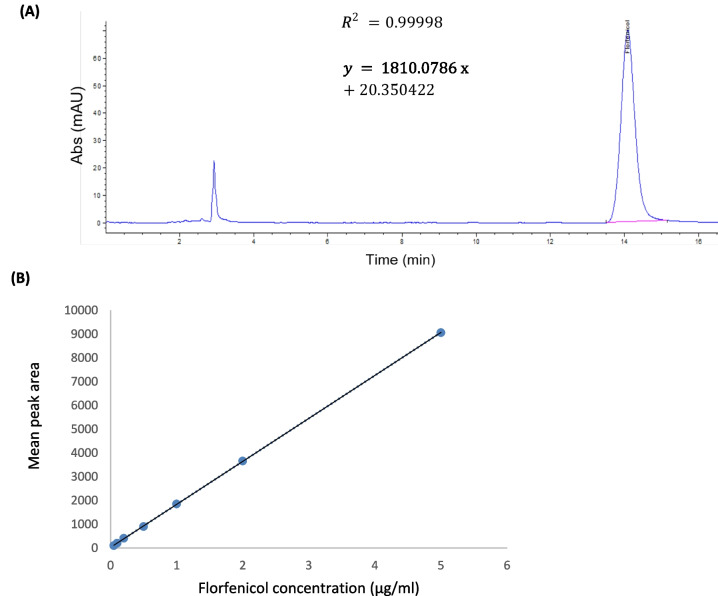
Florfenicol quantification in plasma using HPLC. **A** Chromatogram of 1 µg/ml standard florfenicol in plasma detected at 224 nm. **B** Calibration curve generated using standard florfenicol concentration measured using HPLC

HPLC was employed to determine the florfenicol concentration in chicken-derived plasma following oral administration (Fig. 5). The calibration curve, established using standard florfenicol concentrations ranging from 0.05 to 5 µg/ml in chicken plasma, demonstrated a linear correlation coefficient exceeding 0.999.

**Fig. 6:**
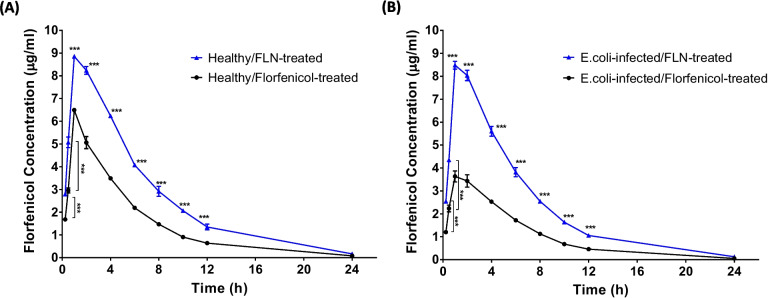
Concentration of florfenicol in plasma collected from **A** healthy chicken or **B** *E. coli*–infected chicken following single oral administration of 30 mg free or niosome-contained florfenicol/kg body weight. Data represent mean ± standard deviation (S.D.). (*n* = 6). *** *P* < 0.001

The pharmacokinetic profiles of both free and niosome-incorporated florfenicol in broilers were assessed. The mean plasma concentrations of florfenicol in healthy control broiler chickens or those infected with *E. coli* following oral administration of free or niosome-encapsulated florfenicol at a dosage of 30 mg florfenicol/kg body weight are shown in Fig. 6. The therapeutic efficacy of florfenicol relies on surpassing the MIC within a specified timeframe [53]. Notably, 12 h following their oral administration to *E. coli*–infected chickens, FLN induced significantly higher florfenicol plasma concentrations (1.05 ± 0.08 µg/ml) than those observed after free florfenicol administration (0.46 ± 0.03 µg/ml). Although these plasma concentrations remained below the MIC values detected in this study for *E. coli* (4 µg/ml) and *S. aureus* (2 µg/ml), they exceeded the values previously reported for florfenicol against *Pasteurella* isolated from chicken and other bacterial strains isolated from cows that were 0.312 µg/ml and 2 µg/ml, respectively [54, 55].

Higher plasma florfenicol levels were achieved through the oral administration of FLN rather than free florfenicol. After the oral administration of a single dose of either florfenicol or FLN at 30 mg/kg body weight, the peak plasma levels for both free florfenicol and FLN were attained 1-h post-administration. The drug was detectable for a duration of 24 h in both healthy and infected broilers following the oral administration of either free florfenicol or FLN. Pharmacokinetic parameters describing the disposition of florfenicol in control healthy and infected broilers following oral administration are summarized in Table 3.

**Table 3:** Pharmacokinetic parameters of free and niosome-contained florfenicol in apparently healthy and *E. coli* –infected chickens after oral administration at a dose of 30 mg florfenicol/kg body weight

**Parameter**	**Unit**	**Healthy/florfenicol-treated**^**a**^	***E. coli*–infected/Florfenicol-treated**^**a**^	**Healthy/FLN-treated**^**a**^	***E. coli*–infected/FLN-treated**^**a**^
A	μg/ml	9.54 ± 0.87	6.02 ± 0.40	13.75 ± 1.62	14.63 ± 1.19
Kab	h^−1^	1.56 ± 0.01	1.35 ± 0.01	1.41 ± 0.04	1.26 ± 0.02
Absorption *T*_1/2_	h	0.44 ± 0.03	0.51 ± 0.02	0.49 ± 0.13	0.55 ± 0.02
B	μg/ml	7.29 ± 0.46	4.20 ± 0.14	9.75 ± 0.58	10.23 ± 0.44
Kel	h^−1^	0.17 ± 0.01	0.21 ± 0.01	0.17 ± 0.01	0.19 ± 0.01
Elimination *T*_1/2_	h	4.08 ± 0.26	3.23 ± 0.01	4.08 ± 0.12	3.65 ± 0.13
C_max_	μg/ml	5.72 ± 0.05	3.68 ± 0.11	8.80 ± 0.04	8.39 ± 0.06
T_max_	h	1.56 ± 0.02	1.69 ± 0.01	1.68 ± 0.01	1.73 ± 0.02
AUC_0-t_	µg/h/ml	34.67 ± 0.20	26.24 ± 0.74	61.64 ± 0.39	55.33 ± 0.30
AUC_0-inf_	µg/h/ml	35.59 ± 0.28	26.66 ± 0.74	63.12 ± 0.44	56.40 ± 0.35
AUMC	µg/h/ml	215.46 ± 5.42	165.71 ± 4.31	410.74 ± 7.34	342.64 ± 6.81
MRT	h	6.05 ± 0.11	6.22 ± 0.01	6.51 ± 0.08	6.07 ± 0.10

^a^Results represent the mean value ± standard error of the mean (*n* = 6)

**Fig. 7:**
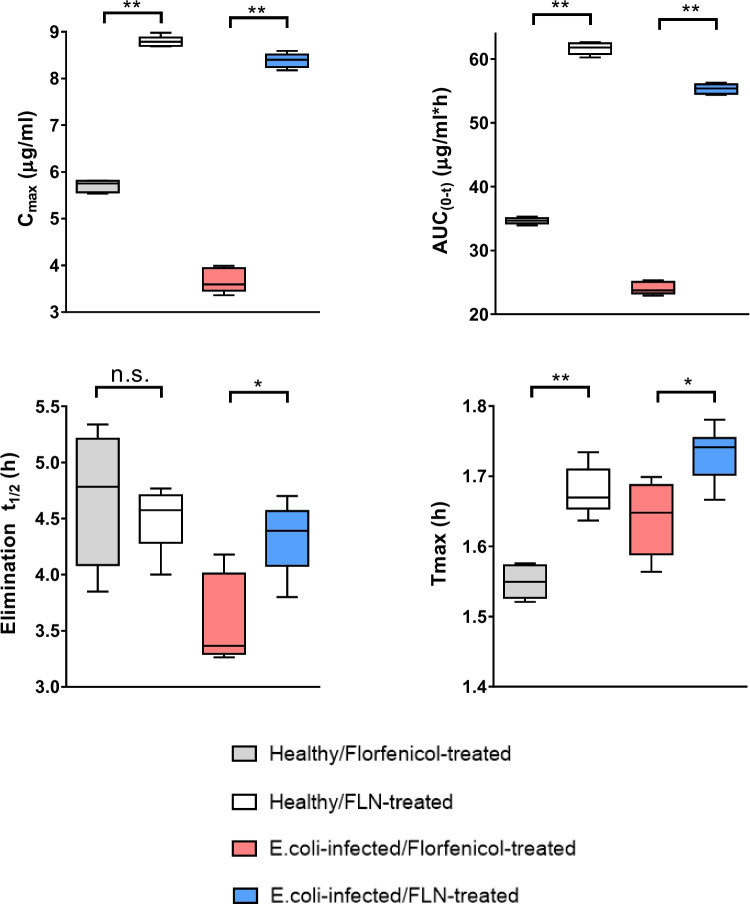
Box plot of *C*_max_, *T*_max_, AUC_(0-t)_, and elimination *T*_1/2_ of free or niosome-incorporated florfenicol post the oral administration to healthy or *E. coli*–infected chicken. The horizontal line inside the box represents the median value. The whisker below and above the box are drawn down to the 10th percentile and up to the 90th percentile, respectively. Statistical analysis was carried out using Mann–Whitney test. *p < 0.05, **p < 0.01

The results obtained from our study revealed significant differences in the pharmacokinetic profiles of florfenicol following oral administration to *E. coli*-infected broilers in the free or niosome-loaded forms. The maximum plasma concentration (C_max_) of florfenicol was notably higher following oral administration of FLN (8.39 ± 0.06 μg/ml) compared to free florfenicol (3.68 ± 0.11 μg/ml) (Fig. 7). This substantial increase in C_max_ highlighted the enhanced systemic delivery achieved through the niosomal formulation. Furthermore, a significantly prolonged elimination half-life (t_1/2_) following oral FLN administration compared to free florfenicol was observed. The prolonged elimination t_1/2_ suggested that FLN persisted in the body for a longer duration, potentially leading to a sustained therapeutic effect. In addition to the extended elimination t_1/2_, FLN also exhibited a significantly higher AUC_0-t_ compared to free florfenicol after the administration of a single oral dose to *E. coli*-infected broilers (Fig. 7). The marked difference in AUC_0-t_ suggests that the niosomal formulation significantly enhanced the bioavailability of florfenicol. Collectively, our findings demonstrated the potential of the niosomal formulation to improve the systemic delivery and bioavailability of florfenicol that could have implications on its therapeutic efficacy in clinical settings.

No adverse effects were noted in broilers during or after the oral administration of FLN. The pharmacokinetics and pharmacodynamics of nano-pharmaceutical formulations in poultry species were assessed in a limited number of studies [56–58]. The results obtained indicated that plasma concentrations of florfenicol were significantly lower in diseased broilers than in their healthy counterparts at the same time following the oral administration of either free or niosome-incorporated florfenicol. This observation could be attributed to the more rapid extravascular distribution of either free florfenicol or FLN in diseased broilers compared to healthy ones. The rapid and extensive distribution of antimicrobial drugs in diseased tissues was previously reported for florfenicol in chickens [55] and ducks [59], as well as for other antibacterial drugs in chickens [60] and mammals [61, 62].

The C_max_ and T_max_ determined following oral administration of free florfenicol to healthy broilers were 5.72 ± 0.05 µg/ml and 1.56 ± 0.02 h, respectively, which were consistent with values previously reported for a water-soluble formulation of florfenicol in healthy chickens [63]. Antibiotics with concentration-dependent activity relay on the presence of high blood concentrations to induce their antibacterial effects, rendering high serum concentration essential [64]. Conversely, antibiotics characterized by time-dependent activity, such as florfenicol antibiotics, depend on the duration during which the antibiotic plasma concentration remains above the MIC for their bacterial-killing abilities [58, 65, 66]. Hence, the evaluation of florfenicol's therapeutic efficacy could be conducted through the assessment of time-dependent concentration changes rather than fluctuations in peak concentrations. In this regard, time-dependent concentration alterations of the two forms of florfenicol, namely free florfenicol and FLN, were examined. It was observed that FLN reached a higher C_max_ that decreased over an extended period of approximately 24 h post-oral intake, maintaining a higher concentration compared to free florfenicol. These observations demonstrated the ability of the niosomal formulation to improve the pharmacokinetic profile of florfenicol.

The intestinal epithelium could be traversed by nanoparticles through either the paracellular route employing a passive diffusion mechanism or the transcellular route via passive diffusion, endocytosis, or carrier-mediated transport [58, 67, 68]. Nanoparticles falling within the particle size range of 10 to 1000 nm could cross the intestinal mucosa through the endocytosis mechanism [69]. The absorption of amoxicillin-loaded negatively charged nanoparticles, with a mean size of 513.96 ± 19.46 nm, through the gastrointestinal tract was previously reported by Güncüm et al*.* [58]. It may be suggested that the fractions of florfenicol that gained access to the systemic circulation following oral administration were a result of the absorption of both intact drug-loaded nanoparticles and released drug through the gastrointestinal barrier. Furthermore, the high plasma concentrations detected after the administration of FLN could be attributed to the release of the drug loaded in the niosomal nanoparticles that were adhered to the mucosal surface.

The results indicated that the incorporation of florfenicol into niosomes significantly increased the *C*_max_ and AUC of the loaded florfenicol in *E. coli*–infected birds. This increase could be attributed to the capability of the florfenicol-loaded nanoformulation to enhance the oral absorption of the incorporated drug by extending gastrointestinal residence time or facilitating epithelial penetration through mucosal adhesion. Additionally, this effect could be linked to the niosomal capacity to protect the loaded drug from enzymatic and non-enzymatic degradation within the gastrointestinal tract [58]. It was previously suggested that nano-pharmaceutical formulation could enhance the oral bioavailability of lipophilic drugs through the enhancement of the loaded drug solubility and the provision of a larger interfacial area for drug absorption [70–72].

Due to the previously reported variable sensitivity of florfenicol against various bacterial strains, ranging from 0.5 to 6 µg/ml [63], the findings of this study cannot be generalized against other bacterial strains [10, 16]. The rational dosage regimen for the florfenicol nanoformulation, based on the pharmacokinetic/pharmacodynamic approach, would primarily rely upon the time course of florfenicol’s effect, i.e., the pharmacodynamic properties guided by the time-dependent pharmacological activity of florfenicol. The results presented in this study suggest that the therapeutic concentration could be maintained via the oral administration of repeated FLN doses at 30 mg florfenicol/kg body weight every 12 h to *E. coli*–infected chickens. Nevertheless, the clinical application of FLN on broiler chickens demands the assessment of the niosomal biocompatibility via the conductance of toxicological studies.

### Toxicity and pharmacological evaluation of FLN

The results of the acute toxicity study showed that the LD50 values for both free and niosome-incorporated florfenicol exceeded 5 g/kg body weight, as all the animals that were orally administered doses of 2 and 5 g of florfenicol/kg body weight survived. In addition, no signs of toxicity were detected in any of the animals throughout the observation period. These observations suggest that the FLN formulation falls within the non-toxic category, as defined by established toxicity criteria for chemical substances [73]. This could be attributed to both the inherently low toxicity of florfenicol itself and the excellent biocompatibility of the surfactant employed [74].

**Fig. 8:**
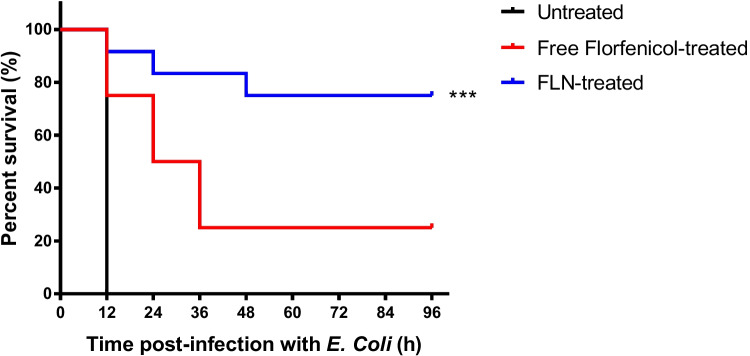
Mortality protection. Percentage survival of lethal dose–infected mice treated with a single administration of 40 mg free or niosome-contained florfenicol/kg body weight (*n* = 12). ***p < 0.001

To evaluate the FLN-mediated pharmacological effect in vivo, the mortality protection percentage was evaluated based on the survival rates of *E. coli*–infected mice treated with free or niosome-incorporated florfenicol. The results obtained indicated that all the mice in the control negative group, which received distilled water, experienced mortality within 24-h post-infection with lethal *E. coli* dose. Furthermore, treating the infected groups with free or niosome-encapsulated florfenicol at florfenicol dose of 20 mg/kg failed to induce mortality protection, as the treated mice did not survive. This observation may be attributed to the sub-therapeutic drug concentrations in the bloodstream of the infected animals, potentially arising from the rapid drug elimination from the body [75]. However, at a florfenicol dose of 40 mg/kg, FLN-treated mice showed significantly higher survival percentage (75%) than the free florfenicol-treated mice (25%) (Fig. 8). The heightened efficacy can be ascribed to the significantly higher relative bioavailability of FLN compared to free florfenicol, as demonstrated in this study.

## Conclusion 

Florfenicol was incorporated into a nanoformulation using self-assembled niosomal carriers. The selection of the optimal formulation parameters was based on pre-formulation studies. Florfenicol-incorporated niosome formulation exhibited favorable physicochemical attributes, improved oral bioavailability, and low toxicity. The niosomal formulation augmented the antibacterial activity of the encapsulated florfenicol against *E. coli* and *S. aureus*, as evidenced by reduced MIC values. Furthermore, in vivo studies revealed that the niosomal florfenicol displayed significantly higher therapeutic efficacy than the free florfenicol form. Collectively, these findings illustrate the potential capacity of niosomes to significantly improve the loaded cargo’s therapeutic outcome in a safe and effective manner.

## Data Availability

The datasets generated during and/or analyzed during the current study are available from the corresponding author on reasonable request.
